# Post-Translational Modifications Aid Archaeal Survival

**DOI:** 10.3390/biom10040584

**Published:** 2020-04-10

**Authors:** Ping Gong, Ping Lei, Shengping Wang, Ao Zeng, Huiqiang Lou

**Affiliations:** 1 Hunan Institute of Microbiology, Changsha 410009, China; fabyoo@126.com (P.L.); fightingbio@163.com (S.W.); victorypro@163.com (A.Z.); 2 State Key Laboratory of Agro-Biotechnology and Beijing Advanced Innovation Center for Food Nutrition and Human Health, College of Biological Sciences, China Agricultural University, No.2 Yuan-Ming-Yuan West Road, Beijing 100193, China

**Keywords:** extremophiles, modification, protein, adaptation, stability

## Abstract

Since the pioneering work of Carl Woese, Archaea have fascinated biologists of almost all areas given their unique evolutionary status, wide distribution, high diversity, and ability to grow in special environments. Archaea often thrive in extreme conditions such as high temperature, high/low pH, high salinity, and anoxic ecosystems. All of these are threats to the stability and proper functioning of biological molecules, especially proteins and nucleic acids. Post-translational modifications (PTMs), such as phosphorylation, methylation, acetylation, and glycosylation, are reportedly widespread in Archaea and represent a critical adaptive mechanism to extreme habitats. Here, we summarize our current understanding of the contributions of PTMs to aid in extremophile survival, with a particular focus on the maintenance of genome stability.

## 1. Introduction

### 1.1. A Sketch of Archaea

Archaea were established, in addition to Eukarya and Bacteria, as one of the three domains of life in the 1970s–1990s by Carl Woese and colleagues [1] based on their phylogenetic studies on small subunit ribosomal RNA (rRNA). According to recent reports, there are four superphyla in the classification system: Euryarchaeota, TACK (Thaumarchaeota, Aigarchaeota, Crenarchaeota, and Korarchaeota), Asgard (Heimdallarchaeota, Thorarchaeota, Lokiarchaeota, and Odinarchaeota), and DPANN (9 phyla, including Nanoarchaeota) [2].

On the one hand, Archaea have some characteristics similar to those of both Bacteria and Eukarya. In terms of cell morphology, material, and energy metabolism, Archaea are close to Bacteria. They have a unicellular lifestyle, lack organelles and nuclei, and have a similar cell shape and size. Both possess a similar DNA structure with one circular DNA molecule, extrachromosomal plasmids, and operons. However, when it comes to genetic information processing, e.g., DNA replication, transcription, translation, and repair, Archaea represent a simplified archetype of their eukaryotic counterparts [2,3]. On the other hand, Archaea also have unique properties. The typical bacterial murein is absent in Archaea, which is regarded as one of the key features of bacterial cell walls. It has reported that Archaea could make pseudomurein, which is chemically a peptidoglycan. Moreover, the archaeal membrane lipids are composed of isoprenoid chains ether-linked to sn-glycerol 1-phosphate head groups other than fatty acids ester-linked to sn-glycerol 3-phosphate, as found in Bacteria and Eukaryotes [4,5].

Many Archaea are extremophilic microorganisms, which thrive in various extreme environments: hot, cold, acid, base, saline, high pressure, anoxic, and high radiation [6]. These extreme environments are not only a challenge to the maintenance of archaeal protein structures and function but also threaten the stability of their genomes. As a result, archaeal extremophiles have a myriad of adaptations that keep their cellular proteins active and maintain genomic stability. Corresponding to their various extreme habitats, Archaea are presently partitioned into several branches, including halophiles, psychrophiles, thermophiles, acidophiles, and methanogens. In addition, since the extreme environments on earth are characterized by coexisting stressors, an increasing number of microbial strains isolated from these environments have been found to tolerate multiple extremes [7,8].

As mentioned above, extremophile Archaea have been found to tolerate multiple different extremes, and the branches of Archaea intersect in interesting ways. For instance, alkaliphiles are clustered together with halophiles since the two archaeal groups are both found in saline environments and also have similar genomes. Similarly, thermophiles are grouped with acidophiles because most acidic environments are hot and also because these groups have similar genomes. For the purposes of this review, we have categorized extremophile Archaea into three general groups—thermophiles, halophiles, and methanogens (Table 1).

#### 1.1.1. Thermophiles

Among the physical parameters important for life, the temperature is probably the most studied. Extreme temperatures can devastate the biomolecules of organisms. Thermal vents and hot springs are considered to be some of the most extreme environments on earth, and while in these hostile locations most life would perish, thermophiles and hyperthermophiles are able to thrive. Although the two share similar adaptations to survive in these extremes, they differ in their temperature growth optimum. Hyperthermophiles are able to grow optimally up to 105 °C, whereas thermophiles are classified as growing between 50 °C and 70 °C. With the exception of a few Bacteria (such as Thermotoga), most of the hyperthermophiles found at present belong to the Archaea, covering almost all Crenarchaeota, some Euryarchaeota, and a few other Archaea species [9]. They can usually withstand high temperatures above 80 °C; for instance, the hyperthermophile *Methanopyrus kandleri* can grow at 122 °C and is one of the most heat-resistant organisms known [10]. For convenience, we employ the term “thermophile” to include both thermophiles and hyperthermophiles unless specified otherwise.

To survive at high temperatures, thermophilic Archaea adopt several strategies. For instance, the archaeal membrane and wall may contribute to the thermal stability of the cellular structure. This may be due to unique chemical compositions, ether linkages, lipid side chain branching, and the structural modifications that may occur when the temperature changes [11,12]. It is critical for thermophilic Archaea to maintain stability and protein activity at extreme temperatures, as proteins that lack the necessary adaptations will undergo irreversible unfolding and exposure of the hydrophobic core, which may cause aggregation. Protein stability is determined by many factors including codon composition, the ratio of charged amino acids to uncharged amino acids, ionic interactions, amino acid distribution, solute accumulation, and posttranslational modifications [13,14].

#### 1.1.2. Halophiles

High-salt ecological environments include salt beaches, evaporation ponds, and high-salt basins in the deep sea. These environments have their own unique physical and chemical parameters, such as total salt concentration, pH value, ion composition, and temperature, but high osmotic pressure is the main challenge that microorganisms face in this kind of environment [15]. High salt may do harm to mesophilic microorganisms, which is mainly due to the destruction of cell membranes and enzymes under high osmotic pressure stress, which then inhibits their physiological activities. Halophilic microorganisms are able to inhabit and reproduce in extreme environments with high salt because of their special physiological structures and metabolic regulatory mechanisms.

Halophilic microorganisms are important branches of extremophiles, and their optimal growth can only be obtained in high concentrations of salt. Halophiles are widespread in environments of various salt concentrations, and according to the salt concentration range of optimum growth can be divided into mild halophiles (optimum salt concentration 0.2–0.5 mol/L), medium halophiles (optimum salt concentration 0.5–2.0 mol/L), and extreme halophiles (optimum salt concentration 3.0 mol/L or higher). Many halophilic microorganisms belong to Archaea [16].

#### 1.1.3. Methanogens

Strictly anoxic environments are regarded as some of the most extreme environments on earth. Methanogenic Archaea are organisms able to thrive in such hostile environments where most life would perish. They are widely distributed in strictly oxygen-free environments, such as underground, in coal reservoirs, marine sediments, swamps, and the rumens and the intestinal tracts of animals [17,18]. To adapt to these extreme environments, methanogenic Archaea (methanogens) convert inorganic or organic compounds into methane and carbon dioxide by anaerobic fermentation and at the same time obtain the energy required for growth.

### 1.2. Post-Translational Modifications (PTMs) in Archaea

PTM refers to the process in which individual amino acid residues on proteins are covalently modified after mRNA is translated into protein [19]. In recent decades, thousands of different PTMs have been identified, including glycosylation, acetylation, phosphorylation, and methylation. By chemically linking various modifying groups and by allowing for changes in the molecular composition of the modifying moieties, covalent modifications can endow proteins with new properties in structure, activity, stability, antigenicity, intracellular localization, and interaction with other proteins or with nucleic acids.

It has long been known that in Eukarya and Bacteria, the fraction of post-translationally modified proteins and their importance are significantly different. PTMs are very common in Eukaryotes, while Prokaryotes are thought to harbor very few modified proteins. A growing number of studies have shown that PTMs are widespread in Archaea [20]. Identified archaeal PTMs include phosphorylation, methylation, glycosylation, ubiquitination, sampylation, and acetylation [21]. PTMs have important effects on the physiological and biochemical functions of the modified protein, suggesting that it is of great significance in Archaea [22]. To an extent, these findings help close the evolutionary gap among Archaea and the other domains of life. For example, acetylation was long considered rare in Archaea. Recently, some studies have reported that acetylation is essential for the viability of haloarchaea [23,24]. Moreover, N-terminal protein acetylation is common in Eukaryotes and Archaea but very rare in Bacteria [25]. This represents an example where archaeal molecular biology resembles that of Eukarya rather than Bacteria.

Extremophiles are microorganisms that can thrive in the most hostile environmental conditions on this planet. Although Archaea express proteins that enable them to adapt to such habitats, PTMs have the advantage of enabling organisms to respond rapidly to changing environmental conditions, such as the depletion of nutrients or changes in abiotic factors such as temperature. This allows cells to change the properties of their current proteome in a way that ensures adaptation and suits their current lifestyle without relying on the synthesis of new proteins. Indeed, archaeal proteins are able to remain correctly folded and functional in the face of extremes of salinity, temperature, and other adverse physical conditions that would generally lead to protein denaturation, loss of solubility, and/or aggregation. Although in most cases, the mechanism for PTM of a particular archaeal protein remains unclear, PTMs have been shown to help archaeal proteins overcome the challenges presented by their surroundings. In the following review, we focus on PTMs and their role in archaeal adaptation to extreme environments.

## 2. PTMs in Environmental Adaption of Archaeal Extremophiles

Because of their unique evolutionary status, distribution range, unique growth environments, and high diversity, Archaea have become very important subjects in biological and biotechnical research [26]. It is of great significance to study the interaction between these organisms and their extreme environments. In this review, we will restrict our emphasis to PTMs and their roles in environmental adaption of archaeal extremophiles.

Up to now, a number of proteome-scale PTM studies have been carried out in thermophilic Archaea. Previous examinations of several archaeal genomes have uncovered the widespread presence of various ostensibly “bacterial” and “eukaryotic” protein kinase and phosphatase paradigms [27]. A total of 1318 phosphorylation sites have been identified through the analysis of 540 different proteins in *Sulfolobus solfataricus*, and these phosphorylated proteins cover almost all proteins with essential functions, such as DNA helicase, primase, topoisomerase, ATPase, and transcription factors [21]. Independently, in *Sulfolobus acidocaldarius*, 801 phosphorylated proteins have been found, most of which belong to archaeal clusters of orthologous genes (arCOGs) [22]. By contrast, other reported archaeal phosphoproteomes from extreme halophile archaea show only 81 sites of phosphorylation in *Halobacterium salinarum* and nine phospho-sites in *Haloferax volcanii* [28,29]. Moreover, it has been found that there are 2518 methylation sites in 872 proteins (accounting for more than half of the identified proteins) in *Sulfolobus islandicus* that are involved in DNA transcription, DNA repair, cell division, cell cycle regulation, signal transduction, and other processes [30]. In addition, an investigation of the *Thermoproteus tenax* proteome revealed widespread modification with 52 methyl-lysines in 30 different proteins; lysine methylation is relatively rare and is catalyzed by sequence-specific lysine methyltransferases in Eukarya and Bacteria [14]. Moreover, methylation and N-terminal acetylation of endogenously purified crenarchaeal RNA polymerase from *Sulfolobus shibatae* and *S. acidocaldarius* have been examined by combined collision-induced dissociation (CID) and electron-transfer dissociation (ETD) mass spectrometry analysis. As a result, 20 and 26 methyl-lysines for *S. shibatae* and *S. acidocaldarius*, respectively, have been identified, and a high-confidence dataset for the mapping of methylation and acetylation sites in both *Sulfolobus* species has been generated [31]. Recently, a proteomic analysis of a wild-type *Sulfolobus islandicus* strain and its mutant derivative strains lacking acetyltransferase or Nt-acetyltransferase revealed a total of 1708 Nε-acetylated lysine residues in 684 proteins (26% of the total proteins), and 158 Nt-acetylated proteins (44% of the identified proteins) have been found in *S. islandicus*. Additionally, consistent with the growth phenotype, the cellular levels of proteins involved in cell division and cell cycle control, DNA replication, and purine synthesis were significantly lowered in the mutant compared to the parental strain [32]. These results suggest that PTMs widely exist in thermophilic Archaea and might contribute to their adaptation to harsh living conditions.

### 2.1. Methylation

Protein methylation is the process of transfer of methyl to proteins from active methyl compounds such as S-adenosylmethionine. In Archaea, the methylated proteins cover many proteins with essential functions that are involved in DNA transcription, DNA repair, cell division, cell cycle regulation, signal transduction and other processes [30]. Protein methylation plays roles in archaeal adaptation to extreme environments by various mechanism, for instance, increasing the stability of proteins, affecting the activity of proteins and regulating interactions between modified proteins and binding partners.

For thermophilic Archaea living at extreme temperatures, the most important thing is to maintain the thermostability and function of proteins—in particular, proteins necessary for survival—and PTMs have increasingly been found to be involved in this process. Among all PTMs, methylation is perhaps the most frequently reported in thermophilic Archaea. To ensure effective methylation, there is a need for efficient catalytic enzymes and sufficient methyl donors. Methionine adenosyltransferase (MAT) plays a key role in the biogenesis of the sulfonium compound S-adenosylmethionine (AdoMet), a crucial methyl donor. It has been revealed that methionine adenosyltransferase from the hyperthermophilic archaeon *Pyrococcus furiosus* (PfuMAT) has an optimum temperature of 90 °C and is characterized by remarkable thermodynamic stability (T_m_, 99 °C), kinetic stability, and resistance to guanidine hydrochloride-induced unfolding. These kinetic features of PfuMAT contribute to the efficient catalysis for AdoMet synthesis in the hyperthermophilic archaeon [33]. In contrast, the MAT in *S. solfataricus* has been reported to have the ability to produce a range of differentially alkylated AdoMet analogs in the presence of nonnative methionine analogs and ATP so as to provide more methyl donors [34]. The adequate supply of methyl donors may explain why methylation is so frequent in thermophiles, and also implicate the importance of methylation for their survival.

Methylation not only increases the thermal stability of proteins but also affects the activity of proteins themselves. It has been found that in *S. solfataricus*, methylated β-glycosidase possesses a greater resistance to protein aggregation and denaturation compared with unmethylated recombinant protein at physiological pH. It is probably due to the significant differences of surface hydrophobicity between the two forms of the protein [35]. Baumann et al., by mass spectrometry, found a methylation modification of the chromatin protein Sso7d, and the degree of protein methylation affected the growth temperature, indicating that the methylation is related to the heat shock reaction and protein stability by strengthening binding to double-stranded DNA to prevent the modified protein from thermal denaturation [36]. McAfee et al. reported that its orthologous protein, Sac7d, in *S. acidocaldarius* was methylated as well. Through differential scanning calorimetry, they found that the reversible unfolding of natural Sac7d is at a Tm value of 100 °C and that of recombinant Sac7d is at 92.7 °C [37]. These results indicate that methylation augments protein thermal stability, although the mechanism needs to be investigated.

Methylation also occurs in other Archaea. According to the substrates, there are three main methane biosynthesis pathways in methanogens, of which acetic acid as a substrate accounts for more than 60% of natural methane synthesis, hydrogen and carbon dioxide as substrates for 30%, and methylated compounds as raw materials for less than 10%. Methyl coenzyme M is formed in three main pathways under the catalysis of methyl coenzyme M reductase. For instance, when acetic acid serves as a substrate, it is first phosphorylated to acetylphosphate and then converted to acetylcoenzyme A. Methyl tetrahydromethyltrexate and a molecule of carbon monoxide are formed under the catalysis of acetylcoenzyme A decarboxylase. Carbon monoxide forms carbon dioxide as catalyzed by carbon monoxide dehydrogenase. The synthetic pathways of carbon dioxide and hydrogen form methyl coenzyme M through a series of electron transfer processes, and the subsequent steps are the same as those of acetic acid. With methyl compounds as the substrate, methyl coenzyme is formed by transferring a methyl group to HS-CoM via catalysis of a key methyltransferase, tetrahydromethanopterin S-methyltransferase (Mtr) [38,39]. There are two homologues of the mtrA gene encoded in methanogenic Archaea from Methanomicrobiales and Methanococccales, one of which is respectively fused with the mtrG and mtrF genes [40]. It follows that methyl coenzyme M is the key component of the methanogenic pathway in that it catalyzes the final reaction in the release of methane, which is formed by methyl transfer from various sources to reduced coenzyme M [41]. Analysis of the crystal structure of the enzyme from *Methanobacterium thermoautotrophicum* shows that the α subunit of the hexameric enzyme possesses five modified amino acid residues, all of which are located near the active site. The four methylated amino acids in methyl-coenzyme M reductase are from the transfer of methyl groups of methionine. In terms of function, methylation of His-257, which participates in substrate binding, might affect the substrate affinity of the enzyme [42,43]. Additionally, methylated tetraethers are produced by *Methanothermobacter thermautotrophicus* in varying proportions depending on growth conditions, implying that methylation could be an adaptive mechanism to regulate cellular function [44].

In addition, methylation has been found to be involved in the stress response of *H. salinarum*. In the process of phototropism, the sensors HtrI and HtrII are methylated to regulate the lifecycle of phototactic signals. When the concentrations of extracellular histidine, aspartic acid, and glutamic acid change, cytoplasmic HtrI is methylated. There is also methylation of the membrane binding receptor HtrIII in aerobic respiration [45]. Although the regulatory mechanisms are obscure, it can be seen that PTM is employed in stress responses by halophilic Archaea.

### 2.2. Phosphorylation

Protein phosphorylation refers the process of transferring ATP phosphate groups to amino acid residues catalyzed by protein kinases. Phosphorylation plays an important role in the regulation of protein activity and function. Reversible protein phosphorylation is also an important mechanism of signal transduction that enables cells to rapidly respond to environmental changes by controlling the functional properties of modified proteins. In Archaea, these phosphorylated proteins cover almost all proteins with essential functions, such as DNA helicase, primase, topoisomerase, ATPase, and transcription factors [21,22].

Thus far, phosphorylation modification has been found on His, Asp, Ser, Thr, Tyr, Cys, Lys, and Arg of eukaryotic proteins [46], while that on Ser, Thr, Asp, His, and Tyr has been found in Archaea [47,48,49]. Ser/Thr and Tyr phosphorylation in Eukaryotes and Archaea is carried out by specific eukaryotic protein kinases (PKs), called Hanks-type kinases (also named ePKs). Up to now, there are seven major clusters of Hanks-type PKs characterized. These are called the tyrosine kinase (TK) group; the tyrosine kinase-like (TKL) group; the calcium and calmodulin-regulated PKs (CAMK) group; PK A, G, and C families (AGC) group; the cycline-dependent/mitogenactivated/glycogen synthase/cycline-dependent like PK (CMGC) group; homologs of yeast STE7, STE11, and STE20 PKs (STE) group; and the cell kinases (CK1) group [50,51]. Although detailed classification of ePKs has not been performed in Archaea, it was found that many Archaea contain at least one ePK [52]. In Euryarchaeota, phosphorylation on His and Asp residues is found in a certain type of regulatory system, the two-component signal transduction systems (TCS), as well as that on Ser and Thr by Hanks-type protein kinases. Instead, Crenarchaeota could only depend on Hanks-type protein phosphorylation due to the lack of TCS [47]. Contrary to the low proportion of Tyr phosphorylated proteins in Eukaryotes, there is a large number of Tyr phosphorylation in *S. solfataricus*. The modified proteins are involved in nearly all core metabolic processes, such as transcription, translation, DNA replication and ATP synthesis [21,49]. Although the phosphorylation of Tyr is very common in Archaea, especially in *Sulfolobus*, so far, no Hanks Tyr protein kinases or bacterial Tyr protein kinases (BY-kinases) has been reported to phosphorylate Tyr in Archaea, indicating the presence of undetected protein kinases that phosphorylate Tyr in Archaea.

Archaellum (formerly flagella) is the motility structure of Archaea [53]. It has report that phosphorylation has taken part in the the expression of archaellum widespreadly. The protein kinase ArnS (Archaellum regulatory network S) plays an essential role in the precisely controlled expression of archaellum components during starvation-induced motility in *S. acidocaldarius*. Although the archaellum could be assembled seemingly, deletion of arns results in reduced motility suggesting that archaellum function is affected [54]. A transcription factor ST0829 in *Sulfolobus tokodaii* can bind to the promoter (ST2519p) region and the gene encoding archaellum protein. While Ser/Thr protein kinases ST1565 inhibit the interaction between ST0829 and ST2519p by phosphorylation, which regulates archaellum protein expression and cell motility eventually [55]. Protein ArnA and ArnB can inhibit the expression of archaellin encoding gene FLAB (Flagellum B) in Archaea. After knockout of both genes, the FlaB content increases and the cell movement ability also increases. Nevertheless, the functions of ArnA and ArnB are jointly regulated by Ser/Thr protein kinase ArnC, ArnD, and Ser/Thr phosphatase, which in turn control the content of Flab in *S. acidocaldarius* [56]. Additionally, an activator of archaellum expression ArnR in *S.solfataricus* is phosphorylated in vivo, suggesting that protein phosphorylation is possibly involved in regulate the movement of Archaea by regulating the expression of archaellum [21].

The halophilic archaeon *Methanohalophilus portucalensis* is the model species to study the mechanism of osmotic pressure regulation. It has been found that 26% of its proteins are involved in the biosynthesis of methane and osmotic pressure regulators by mass spectrometry. Glycine sarcosine N-methyltransferase (GSMT) is a critical enzyme in osmotic pressure regulation of *M. portucalensis* since it can catalyze the synthesis of a vital regulator, betaine. Phosphorylation of Thr68 in GSMT is critical for its methyltransferase activity, because this modification probably shortens the distance between the catalytic center and S-adenosylmethionine [57].

Besides, phosphorylation modification is also involved in the rapidly respond to environmental changes in halophilic Archaea. Once the salt concentration in the environment is higher than optimal for the growth of halophilic Archaea, the structures of some proteins can unfold to adapt to the new conditions, a process called the “unfolding protein response.” Ire1 kinase is a sensor in this pathway in Eukaryote, and in *H. volcanii*, a kinase, hvIre1, was identified as a homolog to the eukaryotic Ire1p kinase. With the increase of NaCl concentration in the medium, the transcription level of hvIRE1 increases accordingly and then initiates the pathway by phosphorylating its target proteins [58,59].

Meanwhile, phosphorylation modification is required for methanogenic Archaea to thrive in extreme environments. Pyrrole lysine (Pyl) can be inserted into multiple methyltransferases and participate in the synthesis of methane. Several phosphorylation sites at the C-terminus of pyrrole lysyl tRNA synthase in *Methanohalophilus portucalensis* can regulate its ability to recognize tRNA^pyl^, affect the synthesis of Pyl-tRNA^pyl^, and then affect the activity of methyltransferase [57]. After knockout of the tRNA^pyl^ gene in *Methanosarcina acetivorans*, cells lose the ability to synthesize methane [60]. It can be inferred that the phosphorylation of pyrrole lysyl tRNA synthase is important for methane synthesis. Moreover, other studies have shown that the synthesis of methane in *Methanosarcinales harundinacea* is regulated by phosphorylation. A signal molecule, carboxylated acyl homoserine lactone, can be synthesized by FilI kinase, which promotes the transition from a short cell to filamentous growth, with an altered carbon metabolic flux that facilitates the conversion of acetate to methane [61].

### 2.3. N-Glycosylation

Protein glycosylation is the process of attaching sugars to proteins under the catalysis of enzymes. In extreme Archaea, protein glycosylation could contribute to the archaeal adaptation to harsh environments by regulating the function of target protein. Thus far, N-glycosylation is more studied, while little is known about O-glycosylation in Archaea [62].

Many PTMs play roles in the adaptation of Archaea to high salt environments, such as glycosylation, phosphorylation, and methylation. Among them, glycosylation is the most widespread probably. This is probably due to the particular cell wall structure. Many Archaea have an surface layer (S-layer) as their sole cell surface component, which is important in maintaining the morphology of Archaea by forming a porous lattice monolayer that wraps outside the cell [63,64]. Additionally, the isoporous lattices completely covering the cell surface provide Archaea with various selection advantages, including functioning as molecular sieves and ion traps, protective coats, as structures employed in surface recognition and cell adhesion, and as antifouling layers [63]. The S-layer generally contains a number of proteins, and they need to undergo a variety of PTMs from the completion of translation to being integrated into the surface of the cell membrane. Compared with Eukaryotes, the species of monosaccharides in the S-layer proteins of halophilic Archaea are more complex, and the glycosylation sites are more changeable [45,62].

Studies have shown that N-glycosylation of S-layer proteins in halophilic Archaea is closely related to their adaptation to high salt environments. For instance, the S-layer glycoproteins from the extreme halophile *Halobacterium salinarum* exhibit a higher degree of glycosylation than those from the moderate halophile *Haloferax volcanii* [65]. What is more, the glycan moieties of the extreme halophile are highly enriched in sulfated glucuronic acid subunits instead of the neutral sugars found in the moderate halophile. These properties render *H. salinarum* S-layer glycoproteins with drastically increased surface charge densities relative to their *H. volcanii* counterpart. The enhanced negative surface charges are considered to contribute to the stability of haloarchaeal proteins in the face of high salt concentrations [66]. Moreover, after knocking out glycosyltransferases AglB or AglD in the N-sugar chain synthesis pathway in *H. volcanii*, the strain grows slowly in a high-salt environment. This is because the regular arrangement and stability of S-layer proteins are affected [67]. Besides, the N-sugar chain structures and modification sites of the S-layer of *H. volcanii* grown at different salt concentrations are changed [68]. These findings confirm the diversity and adaptability of glycosylation modifications in halophilic Archaea.

N-glycosylation has been reported to play an role in the thermophilic Archaea, and some of them probably is related to the adaption. It revealed that the large S-layer subunit SlaA of *S. acidocaldarius* showed a notably high degree of N-glycosylation averaging one modification per 30–40 amino acid residues [69]. Besides, branched N-glycans are considered as a common feature of thermophilic Archaea, whereas the N-glycans of most other Archaea are linear [69,70,71]. These features might be adaptations to the high-temperature environment of thermophilic Archaea. Specifically, disrupted N-glycosylation interfered with normal *S. acidocaldarius* S-layer structure and motility [72,73]. Moreover, when the salinity of the surrounding medium increased, *S. acidocaldarius* cells lacking Agl3 grew increasingly poorly, which is an enzyme involved in N-glycosylation. It suggests a physiological role for cell surface N-glycosylation in this species. As the mutant cells do not contain sulphoquinovose, which is considered to contribute to the hydrated shell that surrounds the cell by providing negative charges. As a result, compromised S-layer stability affects the growth of the mutant as the NaCl concentration of the growth medium increases [74]. Esterases from thermophilic Archaea have an intrinsically high conformational stability, and the thermophilic enzymes yielded from homologous expression should be better biocatalysts than those obtained from mesophilic expression [75,76]. Protein folding and glycosylation have been reported to contribute to the thermal stability of recombinant feruloyl esterase A (EstA) [77]. Glycosylation has long been identified as an important point in the thermal stability of glycoproteins. There are three main mechanisms contribute to the thermal stability of glycoproteins, all of which aim at limiting the peptide backbone flexibility. First, N-glycans could reduce solvent access to regions of the peptide backbone which are both close to and remote from the glycosylation site. Second, N-glycans are revealed to bind aromatic residues, and this contribute to decreasing protein intrinsic fluorescence intensity. Third, N-glycans are found to stabilize disulfide bonds [78,79,80,81]. Nevertheless, the thermal stability of EstA from *S. islandicus* is independent of PTMs, implying that modifications may have functions other than simply affecting the thermal stability of the protein [13].

### 2.4. Other PTMs

Other PTMs also play roles in the adaptation of Archaea to harsh environments. To maintain the stability and function, disulfide bonds are required for most proteins. In order to adapt to the changing environment, intracellular disulfide-shuffling in hyperthermophiles may occur more frequently [82]. What is more, in order to adapt to a high-temperature environment, the PTM mechanism itself may have corresponding evolution. For instance, covalent flavinylation in the hyperthermophilic Archaea, *Sulfolobus tokodaii*, requires both heat and dicarboxylic acid [83].

As well as glycosylation, other modifications play roles in the adaptation of halophilic Archaea to harsh environments. A systematic analysis of lysine acetylation in *Haloferax mediterranei* identified 1017 acetylation sites in 643 proteins, accounting for 17.3% of the total proteins in this haloarchaeon. Moreover, the acetylation of key enzymes in polyhydroxyalkanoate (PHBV) biosynthesis suggested that acetylation may plays a role in energy and carbon storage [24]. The 26S proteasome is an important complex responsible for protein degradation in the cytoplasm of eukaryal and archaeal cells, which are composed of 20S catalytic core particles (of α- and β-type subunits) and regulatory particles [84,85]. In *H. volcanii*, proteasomes undergo acetylation and methyl-esterification in addition to phosphorylation, and these modifications are reported to influence the assembly and/or the degradation function of 20S proteasome. The ubiquitin-like modification sampylation (small archaeal modifier protein), which targets proteins for the proteasomal degradation, was also identified in *H. volcanii* [86,87].

In addition, a putative acetyltransferase gene, MMP0350, has been identified in *Methanococcus maripaludis*, which affects the proper assembly of both archaellum and pili. This suggests that acetylation may also be involved in the motility regulation of Archaea [88].

## 3. PTMs in Maintaining Extreme Archaeal Genome Stability

The extreme environments in which Archaea live are a challenge to the maintenance of their protein structure and function. In addition, these extreme conditions are also a threat to genomic stability. Several factors, such as high temperature and radiation, seriously threaten genomic stability. High temperatures increase the probability of DNA damage; for instance, the optimum growth temperature of *Pyrolobus* is about 107 °C, at which the extremely high spontaneous mutation rate can result in a single-stranded DNA break every 75 kb per minute [89]. Therefore, for extremophiles, powerful DNA damage protection and repair mechanisms are needed to maintain genetic stability in order to adapt to the various extreme living conditions [89,90]. Similar to those in Eukaryotes, chromosomal proteins and DNA replication and DNA damage repair proteins in Archaea play an important role in this safeguard mechanism.

Eukaryotic chromosomal proteins, including histones, play an important role in the packaging of genomic DNA and affect DNA replication, transcription, and repair [91]. Chromosomal proteins with similar functions are synthesized in Archaea, and can bind to DNA nonspecifically so as to improve its Tm value and help maintain its structure under extreme conditions [92].

Precise DNA replication is required for genome stability, and the process is fundamentally conserved in the three domains of life. It has been demonstrated that archaeal DNA replication is more similar to eukaryotic DNA replication than bacterial DNA replication but is far less complicated than in Eukaryotes [93]. The DNA replication of Archaea can be divided into three processes: initiation, extension, and termination, in which PTMs are involved.

DNA repair is another important process in maintaining genome stability. It is reported that many DNA repair proteins in thermophilic Archaea are similar to those of Eukaryotes [94]. For example, the amino acid sequence identity between the thermophilic archaeal DNA damage repair protein RadA and the eukaryotic repair protein Rad51 is 40% [95]. In a few species, the functions of DNA repair proteins in the Archaea include nucleic acid excision repair and recombination repair. Although archaeal DNA repair proteins are significantly fewer than in eukaryotic cells, it is notable that this simplification does not reduce the DNA damage repair capability [96]. For example, the full-length genome of *P. furiosus* incubated at 95 °C, after being damaged by irradiation to create an average of a double-stranded break (DSB) every 30 kb, can be entirely repaired [94]. In addition to RadA, there are Mre11, Rad50, Rad55, Hje, Hjc, and other eukaryotic recombinant repair protein homologs in thermophilic Archaea [97]. Thus far, researchers have reported that PTMs are involved in maintaining extreme archaeal genome stability.

### 3.1. Methylation

Although PTM of histones was shown not to occur in the methanogenic archaeon *Methanococcus jannaschii* [98]. It was reported that a different degree of lysine methylation modification occurs in the DNA-binding proteins Sis10b, Sac10b, Ssh10b, and Sso10b, and chromosomal proteins Cren7 and Sul7 in hyperthermophilic Crenarchaea [14]. Since lysine methylation is known to enhance protein thermostability, this may be an adaptation to a thermophilic lifestyle. Methylation of lysine could increase the pKa of the side chain, allowing stronger ionic interactions to be formed, and change the hydropathy and solubility of modified proteins [35]. Two chromatin proteins, Cren7 and Sso7d, are consistently undermethylated, whereas other chromatin proteins are unaltered in *Sulfolobus solfataricus* [99]. Interestingly, almost all of the above chromosomal protein modifications are catalyzed by lysine methyltransferase (aKMT), which is a highly conserved crenarchaeal protein with broad substrate specificity [100]. This enzyme catalyzes extensive protein lysine methylation in the hyperthermophile *S. islandicus*, despite its being dispensable for growth. Almost all of its known substrates are nucleic acid-binding proteins, suggesting its involvement in the regulation of chromatin structure and gene expression [101].

Up to now, studies on chromosomal proteins have mostly focused on Alba, Sso10a, Cren7, and Sul7 in hyperthermophilic Archaea [102,103]. The 10b protein (Alba) is a conserved chromosomal structural protein existing in most Archaea, usually in the form of a dimer. The Alba dimer binds to the DNA double-stranded grove and increases the stability of the DNA structure by forming complexes with other protein dimers. PTM of Alba impacts genomic architecture and gene expression. It has been shown that the DNA-binding activity of Alba is regulated by acetylation at K16, which is located at the interface interacting with dsDNA and dsRNA. A NADH-dependent deacetylase, Sir2, changes the chromosome structure by deacetylation of Alba, regulating gene expression [104,105]. Intriguingly, more recent studies have found that K16 of Sso10b and Sis10b are methylated instead of being acetylated, and this modification does not regulate transcription or growth [106]. Conflicting information on the PTM status of K16, combined with the possible role of Alba in binding DNA and RNA and the different abundances of the protein in diverse species, suggest that PTM of Alba members may also be diverse and likely impacts aspects of both RNA and DNA binding. Taken together, it can be seen that PTMs of chromosomal proteins in Archaea are involved in the process of stabilizing the DNA structure by regulating the interaction between them.

Mcm (mini-chromosome maintenance) is an AAA^+^ superfamily protein that has 3′–5′ helicase activity and plays an important role in the initiation and prolongation of DNA replication. It has been reported that a few lysine sites of Mcm, K280, K281, K545, and K546 can be methylated by the methyltransferase aKMT4, which is a homolog of *Saccharomyces cerevisiae* ydot1 in *S. islandicus* [107,108]. Methylation makes the protein perform higher helicase activity at physiological temperature and enhances the protein thermostability. It is perhaps due to the fact that methylation improved the surface hydrophobicity and pKa value of the protein. In addition, methylated Mcm has a longer half-life than the unmethylated protein [108]. These findings indicate that methylation regulates DNA replication protein activity as well as thermostability.

The Mre11:Rad50 complex is conserved in Archaea and Eukarya and plays a central role in DSB repair and DNA recombination. The complex has both double-strand-specific 3′–5′ exonuclease activity and single-strand endonuclease activity, which is required to bind DNA ends and hold the complex in close proximity to favor the nuclease activity [109]. Kish et al. found that under normal physiological conditions, Rad50 contains 37 methylated lysine residues, accounting for 30% of all Rad50 lysine residues, and Mre11 has 8 methylated sites, accounting for 30% of all Mre11 lysine residues in *Sulfolobus acidocaldarius* [52]. In addition to the methylation of lysine residues, both proteins contain glutamic methylation modification sites in *S. acidocaldarius*. Several methylated lysines on Mre11 and Rad50 are located in the region where they interact, such as the coiled-coil structure of Rad50 that mediates its interaction with other proteins, including Mre11. Lysine methylation in this structure may regulate the interaction of two spiral structures that may affect the interaction between the proteins. In addition, more methylated aspartic acid and glutamic acid residues appear in Mre11:Rad50 complexes in the process of DNA damage repair when cells are treated with gamma-ray [52]. These results show that protein methylation is involved in the repair of DNA damage.

### 3.2. Phosphorylation

In the presence of DNA damage, Eukaryotes may activate the DNA damage checkpoint pathway to initiate downstream DNA repair. DNA damage repair is a typical phosphorylation signal transduction pathway with core components, including a sensor, transduce kinase, and effect kinase [110]. Although no similar pathway has been found in Archaea so far, some studies have shown that the expression of protein kinases in some Archaea changes in the presence of DNA damage, which suggests that phosphorylation may play a role in the response to DNA damage.

All sequenced Archaea genomes contain the RIO gene, including four types: RIO1, RIO2, RIO3, and RIOB. Studies in Eukaryotes have found that Rio kinase is involved in ribosome synthesis, chromosome stability maintenance, and the progression of the cell cycle [111]. UV irradiation can upregulate the expression of RIO1 in *S. solfataricus* [112]. mRNA levels of RIO1 in *P. furiosus* are upregulated under gamma-ray treatment, which may lead to a higher survival rate [113]. Furthermore, when treated with the DNA alkylating agent methyl sulfonate, the level of Rio1 protein decreases, whereas that of another protein kinase, SiRe_2600, increases in *S. islandicus* [114]. According to these results, we can infer that Rio kinase is involved in the DNA damage response, but the specific mechanism is not clear. Cdc6 is a replication initiation protein that recognizes and binds to the origin of DNA to initiate replication. Cdc6 has been shown to autophosphorylate at serine residues in *M. thermautotrophicum*. This autophosphorylation activity may affect its ability to interact with DNA [115].

### 3.3. Other PTMs

Finally, it is worth emphasizing that other types of PTMs may contribute to genome integrity as well. Apart from the acetylated Alba protein mentioned above, it has been shown that Cdc6, a replication initiation protein, is acetylated in *H. mediterranei,* and mutation of the acetylated site of Cdc6 destroys the autonomous replication sequence (ARS) activity of its adjacent origin, oriC [24].

## 4. Conclusions

Here, we attempt to review the PTMs of archaeal proteins that contribute to adaptation to extreme environments (Table 2). Additionally, PTMs that maintain archaeal genomic stability under extreme conditions are also summarized. PTMs can modulate the physicochemical and biological properties of archaeal proteins including thermal stability, folding, oligomerization, activity, subcellular localization and interactions with other proteins, nucleic acids or small molecules. The detailed mechanisms by which PTMs promote archaeal adaptation to extreme environments is another treasure waiting to be uncovered. Finally, it is worth pointing out that not all Archaea are extremophiles. It will therefore be of great interest to compare the PTMomics of extremophiles with that of their close mesophilic relatives in the future.

## Figures and Tables

**Table 1:** Growth characteristics of archaeal extremophiles discussed in this review.

Archaeal Extremophiles	Main Species	Growth Characteristics	Habitats
Thermophiles	*Sulfolobus islandicus, Sulfolobus shibatae, Sulfolobus acidocaldarius, Pyrococcus furiosus, Sulfolobus solfataricus*	Most optimal temperatures between 50–70 °C; a few hyperthermophiles can grow at 122 °C	Thermal vents, hot springs, volcanic vents
Halophiles	*Halobacterium salinarum, Haloferax volcanii, Haloferax* *m* *editerranei, Methanohalophilus portucalensis*	The optimum salt concentration of medium halophiles for growth is 0.5–2.0 mol/L; the extreme halophiles grow optimally in 3.0 mol/L or higher salt concentrations	Salt beaches, evaporation ponds, high-salt basin in the deep sea
Methanogens	*Methanobacterium thermoautotrophicum, Methanothermobacter thermautotrophicus, Methanohalophilus portucalensis*, *Methanosarcina acetivorans, Methanosarcinales harundinacea, Methanococcus maripaludis*	Distributed in strictly oxygen-free environments	Coal reservoirs, marine sediments, swamps, rumens and intestinal tracts of animals

**Table 2:** Overview of some representative post-translational modifications (PTMs) discussed in this review.

Species	Target Proteins	PTMs	Roles of Modifications	Ref.
*S. solfataricus*	β-glycosidase	Methylation	Resistant to aggregation and denaturation at physiological pH.	[35]
*S. solfataricus*	Sso7d	Methylation	Affects the heat shock reaction and stability of the protein.	[36]
*S. acidocaldarius*	Sac7d	Methylation	Enhances thermal stability.	[37]
*S. islandicus*	Esterases	Glycosylation	Unknown.	[13]
*S. solfataricus*	Cren7	Methylation	Enhances protein thermostability.	[14]
*S. solfataricus*	Alba protein	Acetylation	Reduces the affinity of the protein for DNA; regulates transcription.	[104,105]
*S. islandicus*	Alba protein	Methylation	No effect on growth or transcription.	[106]
*S. islandicus*	Mcm helicase	Methylation	Stimulates helicase activity; enhances thermostability; affects protein-protein interactions; increases the half-life the protein.	[108]
*M. thermautotrophicum*	Cdc6	Phosphorylation	Affects its ability to interact with DNA.	[115]
*H. mediterranei*	Cdc6	Acetylation	Affects the autonomous replication sequence activity of its adjacent origin, oriC.	[24]
*S. acidocaldarius*	Mre11:Rad50 complex	Methylation	May affect the interactions of proteins with other molecules and the functional coordination.	[52]
*H. salinarum* *H. volcanii* …	S-layer	Glycosylation	Increases surface charge density; enhances protein stability.	[65,66,67]
*H. mediterranei*	Polyhydroxyalkanoate (PHBV) enzyme	Acetylation	Unknown.	[24]
*H. volcanii*	26S proteasome	Acetylation; methyl-esterification; phosphorylation	Influences the assembly and/or degradation function of the 20S proteasome.	[86,87]
*M. portucalensis*	Glycine sarcosine N-methyltransferase (GSMT) enzyme	Phosphorylation	Influences the methyltransferase activity.	[57]
*H. salinarum*	Htr sensor	Methylation	Regulates the lifecycle of phototactic signals.	[45]
*M. thermoautotrophicum*	Mcr enzyme	Methylation	Might affect the substrate affinity of the enzyme.	[42,43]
*M. portucalensis*	Pyrrole lysyl tRNA synthase	Phosphorylation	Regulates its ability to recognize tRNA^pyl^, affects the synthesis of Pyl-tRNA^pyl^, is important for methane synthesis.	[57]
*S. tokodaii*	ST0829	Phosphorylation	Regulating the expression of archaellum	[55]
*S. acidocaldarius*	ArnA, ArnB	Phosphorylation	Regulation of archaellum expression	[56]
*S.* *solfataricus*	ArnR1	Phosphorylation	Regulation of archaellum expression	[21]
